# Psychological processes underlying the association between childhood trauma and psychosis in daily life: an experience sampling study

**DOI:** 10.1017/S003329171600146X

**Published:** 2016-07-12

**Authors:** U. Reininghaus, C. Gayer-Anderson, L. Valmaggia, M. J. Kempton, M. Calem, A. Onyejiaka, K. Hubbard, P. Dazzan, S. Beards, H. L. Fisher, J. G. Mills, P. McGuire, T. K. J. Craig, P. Garety, J. van Os, R. M. Murray, T. Wykes, I. Myin-Germeys, C. Morgan

**Affiliations:** 1 Department of Psychiatry and Psychology, School for Mental Health and Neuroscience, Maastricht University, Maastricht, The Netherlands; 2 Health Service and Population Research Department, Centre for Epidemiology and Public Health, Institute of Psychiatry, Psychology & Neuroscience, King's College London, London, UK; 3 Psychology Department, Institute of Psychiatry, Psychology & Neuroscience, King's College, London, UK; 4 Psychosis Studies Department, Institute of Psychiatry, Psychology & Neuroscience, King's College, London, UK; 5 National Institute for Health Research (NIHR) Mental Health Biomedical Research Centre (BRC) at South London and Maudsley NHS Foundation Trust and King's College London, London, UK; 6 MRC Social, Genetic & Developmental Psychiatry Centre, Institute of Psychiatry, Psychology & Neuroscience, King's College London, London, UK; 7 Department of Medicine, Psychiatry Research Group, Catholic University of Leuven, Leuven, Belgium

**Keywords:** Childhood abuse, ecological momentary assessment, first-episode psychosis, mechanism, prodrome, resilience, stress sensitivity, threat anticipation

## Abstract

**Background:**

Evidence has accumulated that implicates childhood trauma in the aetiology of psychosis, but our understanding of the putative psychological processes and mechanisms through which childhood trauma impacts on individuals and contributes to the development of psychosis remains limited. We aimed to investigate whether stress sensitivity and threat anticipation underlie the association between childhood abuse and psychosis.

**Method:**

We used the Experience Sampling Method to measure stress, threat anticipation, negative affect, and psychotic experiences in 50 first-episode psychosis (FEP) patients, 44 At-Risk Mental State (ARMS) participants, and 52 controls. Childhood abuse was assessed using the Childhood Trauma Questionnaire.

**Results:**

Associations of minor socio-environmental stress in daily life with negative affect and psychotic experiences were modified by sexual abuse and group (all *p*_FWE_ < 0.05). While there was strong evidence that these associations were greater in FEP exposed to high levels of sexual abuse, and some evidence of greater associations in ARMS exposed to high levels of sexual abuse, controls exposed to high levels of sexual abuse were more resilient and reported less intense negative emotional reactions to socio-environmental stress. A similar pattern was evident for threat anticipation.

**Conclusions:**

Elevated sensitivity and lack of resilience to socio-environmental stress and enhanced threat anticipation in daily life may be important psychological processes underlying the association between childhood sexual abuse and psychosis.

## Introduction

Over the past decade, evidence has accumulated that implicates childhood trauma in the aetiology of psychosis. Childhood trauma refers to a range of early negative and potentially harmful experiences including sexual, physical and emotional abuse (Morgan & Fisher, 2007). Findings from a modest number of epidemiological studies suggest childhood trauma confers risk of experiencing psychotic symptoms (Morgan & Fisher, 2007; Varese *et al.* 2012). While most studies to date have focused on the role of childhood sexual abuse (Morgan & Fisher, 2007; Varese *et al.* 2012; Matheson *et al.* 2013), and some authors have argued that sexual and physical abuse are potent risk factors (Read *et al.* 2005; Bentall *et al.* 2014), emotional abuse has also been associated with an increased risk of psychosis (Varese *et al.* 2012).

Although we know there is an association between childhood abuse and psychosis, we know little about the psychological processes and mechanisms involved. Current models of psychosis suggest that exposure to trauma in childhood makes individuals more sensitive to subsequent adversity through enhanced stress sensitivity and threat anticipation (Myin-Germeys *et al.* 2001; Morgan & Hutchinson, 2010; Morgan *et al.* 2010, 2014; Howes & Murray, 2014). This type of model is supported by an experience sampling study in general practice, which showed that individuals exposed to childhood physical and sexual abuse reported more intense negative emotional reactions to daily life stress (Glaser *et al.* 2006). Similar results have been found in responses to daily hassles in individuals with depression (Wichers *et al.* 2009) and enduring psychotic disorder (Lardinois *et al.* 2011).

Childhood trauma may increase sensitivity not only to minor stressful events but also to more minor adverse social contexts and experiences later in daily life, including distinctive unpleasant social situations (referred to here as ‘social stress’) (Myin-Germeys *et al.* 2001; Morgan *et al.* 2014), subjective experiences of outsider status (as one specific, potentially relevant form of social stress) (Morgan & Fisher, 2007; Morgan *et al.* 2010; Gevonden *et al.* 2015), and unpleasant neighbourhoods (referred to here as ‘area-related stress’) (Jaffee *et al.* 2007; Kirkbride *et al.* 2014). Further, exposure to adverse and threatening experiences during childhood may lead individuals to anticipate more unpleasant events and threat from their environment to create an enduring sense of threat anticipation (Morgan *et al.* 2010; Bentall *et al.* 2014). The Experience Sampling Method (ESM) provides a context-sensitive approach to study whether childhood trauma may amplify threat anticipation as well as stress sensitivity and, thereby, contribute to the development of psychotic experiences in daily life. However, previous studies have not investigated this in individuals with first-episode psychosis (FEP) compared to individuals with an At-Risk Mental State (ARMS; also known as High-Risk or Ultra-High-Risk states) for psychosis (Yung *et al.* 2005; Fusar-Poli *et al.* 2013*a*), and controls. This would allow us to minimize bias due to the potential effects of illness chronicity and further elucidate the impact of putative psychological mechanisms across different stages in the development of psychosis.

Using data from an experience sampling study of FEP individuals, ARMS individuals, and controls with no personal or family history of psychosis, we aimed to investigate whether elevated stress sensitivity and enhanced threat anticipation are important psychological mechanisms underlying the association between childhood sexual, physical and emotional abuse and psychosis. We previously demonstrated in this sample that elevated stress sensitivity, characterized by intense emotional reactions to event-related stress, social stress, area-related stress and experiences of outsider status, as well as enhanced threat anticipation are important psychological processes in the development of psychotic experiences in daily life across different stages of subclinical, prodromal, and FEP (Reininghaus *et al.* 2016*c*). In the current study, we aimed to investigate whether associations of putative psychological mechanisms (event-related stress, social stress, area-related stress, experiences of outsider status, threat anticipation) with (i) negative affect and (ii) psychotic experiences were modified by prior exposure to childhood abuse (sexual, physical, and emotional abuse) and group (FEP, ARMS, controls). Specifically, we sought to test the following hypotheses: (1) within each group, the magnitude of associations of each psychological mechanism with (i) negative affect and (ii) psychotic experiences is greater in individuals exposed to high levels of abuse *v.* those exposed to low levels of abuse (or, in short, the responses to stress and threat anticipation are more pronounced in those exposed) [first set of hypotheses (H1)]; and (2) the difference in magnitude of associations of each putative psychological mechanism with (i) negative affect and (ii) psychotic experiences (put simply, the difference in responses to stress and threat anticipation) between those exposed to high levels and those exposed to low levels of abuse is greater in (*a*) FEP than in controls, (*b*) ARMS than in controls, and (*c*) FEP than in ARMS [second set of hypotheses (H2)].

## Method

### Sample

We recruited a sample of FEP individuals, ARMS individuals, and controls with no personal or family history of psychosis identified in the Childhood Adversity and Psychosis (CAPsy) study and the London centre of the European Network of National Networks studying Gene-Environment Interactions in Schizophrenia (EU-GEI, 2014). Individuals with FEP were recruited from mental health services within defined catchment areas in South-East London, UK. ARMS individuals were recruited from Outreach and Support in South London (OASIS), a clinical service for people at high risk of psychosis provided by the South London and Maudsley NHS Foundation Trust (Fusar-Poli *et al.* 2013*b*), the West London Mental Health NHS Trust (WLMHT), and a community survey of General Practitioner (GP) practices. Controls were recruited using GP lists and the national postal address file as sampling frames. Inclusion and exclusion criteria for FEP, ARMS and controls are shown in Table 1.

**Table 1.** Inclusion and exclusion criteria for FEP, ARMS, and controls

Sample	Inclusion/exclusion criteria
FEP	
Inclusion criteria	• Aged 18–64 • Resident within the defined catchment areas; presence of a first episode of psychosis [ICD-10 F20–F29, F30–F33 diagnoses (WHO, 1992), based on the OPCRIT system (McGuffin *et al.* 1991; Reininghaus *et al*. 2016a)] • Adequate command of the English language to complete the assessments
Exclusion criteria	• Transient psychotic symptoms resulting from acute intoxication • Psychotic symptoms precipitated by an organic cause
ARMS	
Inclusion criteria	• Aged 18–35 • Presence of an ARMS based on CAARMS^a^ (Yung *et al.* 2005) or the SPI-A (i.e. meeting the at-risk criterion of cognitive-perceptive basic symptoms) (Schultze-Lutter *et al.* 2007, 2010, 2012; Klosterkotter *et al.* 2011; Mills, 2014) • Adequate command of the English language
Exclusion criteria	• Prior experience of a psychotic episode for more than one week as determined by the CAARMS and SCID (First *et al.* 2002) • Previous treatment with an antipsychotic for a psychotic episode • IQ < 60 as measured with an adapted version of the WAIS (Ryan *et al.* 1999; EU-GEI, 2014)
Controls	
Inclusion criteria	• Aged 18–64 • Resident within the same areas as FEP individuals • Adequate command of the English language
Exclusion criteria	• Personal or family history of psychotic disorder (Maxwell, 1992) • Presence of psychotic experiences, measured with the Psychosis Screening Questionnaire (Bebbington & Nayani, 1995) • Presence of an ARMS based on the CAARMS or SPI-A

ARMS, At-Risk Mental State for psychosis; CAARMS, Comprehensive Assessment of At-Risk Mental States; FEP, first-episode psychosis; OPCRIT, Operational CRITeria system; SCID, Structured Clinical Interview for DSM Disorders; SOFAS, Social and Occupational Functioning Assessment Scale; SPI-A, Schizophrenia Proneness Instrument – Adult version; WAIS, Wechsler Adult Intelligence Scale.

a ARMS criteria based on the CAARMS (Yung *et al.* 2005; EU-GEI, 2014): (1) Schizotypal personality disorder plus a recent decline in function [defined as (i) a 30% drop in the SOFAS score (Goldman *et al.* 1992) from premorbid level, sustained for 1 month, and occurring within past 12 months; or (ii) a SOFAS score of ⩽50 for past ⩾12 months]. (2) First-degree relative with psychosis plus a recent decline in function (see above). (3) ‘Attenuated’ positive psychotic symptoms. (4) Brief psychotic episode of less than one week duration that resolves without antipsychotic medication.

### Data collection

#### Socio-demographic characteristics

Data on age, gender, ethnicity, level of education, and employment status were collected using a modified version of the Medical Research Council (MRC) socio-demographic schedule (Mallet, 1997; EU-GEI, 2014).

#### Sexual, physical and emotional abuse

Sexual, physical and emotional abuse were measured using an established self-report measure, i.e. the Childhood Trauma Questionnaire (CTQ; Bernstein & Fink, 1998), which measures the severity continuously with five items for each type of abuse before the age of 16. The CTQ asks participants to rate each item on a 5-point Likert scale (1 = never true, 5 = very often true) and allows computation of mean scores for each type of abuse ranging from 1 to 5 as well as categorical severity scores. Good psychometric properties have been reported for the CTQ in clinical as well as community samples (Scher *et al.* 2001; Wright *et al.* 2001).

#### ESM measures

Data on stress, threat anticipation, negative affect, and psychotic experiences were collected with ESM assessments scheduled at random within set blocks of time (Myin-Germeys *et al.* 2001, 2009; Shiffman *et al.* 2008; Palmier-Claus *et al.* 2011, 2012). Previous ESM research in samples of patients with psychotic disorder (Myin-Germeys *et al.* 2001; Lardinois *et al.* 2011), ARMS individuals (Palmier-Claus *et al.* 2012), and controls (Myin-Germeys *et al.* 2001; Palmier-Claus *et al.* 2012) has demonstrated the feasibility, reliability and validity of the assessment method (Myin-Germeys *et al.* 2009; Palmier-Claus *et al.* 2011). All participants were given an electronic momentary assessment technology device (the PsyMate^®^, www.psymate.eu/) (Myin-Germeys *et al.* 2011). A detailed description of the ESM procedure and measures (Myin-Germeys *et al.* 2001, 2005; Delespaul *et al.* 2002; Corcoran *et al.* 2006; Bentall *et al.* 2008, 2009; Freeman *et al.* 2013) is shown in Table 2 and provided in Reininghaus *et al.* (2016*c*).

**Table 2.** ESM procedure^a^ and measures of stress, negative affect, threat anticipation, and psychotic experiences

Domain	ESM measure
Stress	Event-related and social stress was operationalized as minor disturbances and distinctive unpleasant events and social situations that occur in the natural flow of daily life [based on previous ESM studies, in which good internal consistency and concurrent validity with other stress measures have been reported (Myin-Germeys *et al.* 2001; Palmier-Claus *et al.* 2011)]
Event	Event-related stress was measured with one item asking participants to rate the most important event since the last beep on a 7-point Likert scale ranging from ‘very unpleasant’ (rating of −3) to ‘very pleasant’ (rating of 3) (Myin-Germeys *et al.* 2001). We reversed the coding of this item in order for higher ratings to indicate higher levels of stress (with ratings of −3 (i.e. ‘very unpleasant’) coded as 7 and ratings of 3 (i.e. ‘very pleasant’) coded as 1) (Myin-Germeys *et al.* 2001)
Social	The ESM social stress measure we used consisted of two items to assess moments where an individual's current social environment induces minor stress in the natural flow of daily life [based on previous ESM studies (Myin-Germeys *et al.* 2001)]. Participants were first asked to indicate on a categorical item ‘Who am I with?’ (partner, family, friends, colleagues, acquaintances, strangers, others, nobody) and then asked to rate their current social context on a 7-point Likert scale ranging from ‘not at all’ (rating of 1) to ‘very much’ (rating of 7)) using the following two items: (1) ‘I would prefer to be alone [if with someone]/I would prefer to have company [if alone]’; (2) ‘I find being with these people pleasant [if with someone]/it is pleasant to be alone [if alone]’. The coding of item 2 was reversed and the mean score of these two items computed as a measure of minor social stress in daily life (Cronbach's *α* = 0.62) (Myin-Germeys *et al.* 2001)
Area-related	Area-related stress was assessed by asking participants to rate one item ‘I find being in this neighbourhood unpleasant’ on a 7-point Likert scale
Outsider status	Based on previous research that suggests exposure to social adversity may sensitize individuals to subjective experiences of outsider status as one potentially relevant form of social stress (Morgan & Fisher, 2007; Morgan *et al.* 2010; Gevonden *et al.* 2015), following ratings of current social context, participants were asked to rate one item (‘I feel I am an outsider’) on a 7-point Likert scale to assess experiences of outsider status. The association of this item with the ESM social (*r* = 0.33, *p* < 0.001) and area-related (*r* = 0.35, *p* < 0.001) stress measures indicated the item taps a distinct but related aspect of social stress and, overall, reasonable concurrent validity
Negative affect	We used a 5-item ESM measure for assessing negative affect. This measure asks participants to rate the following items at each entry point on a 7-point Likert scale: ‘I feel anxious’, ‘I feel down’, ‘I feel lonely’, ‘I feel insecure’, and ‘I feel annoyed’ (Cronbach's *α* = 0.86) (Myin-Germeys *et al.* 2001)
Threat anticipation	Our ESM measure of threat anticipation was based on a self-report format used for assessing this mechanism in previous cross-sectional studies asking participants to rate the likelihood of negative events happening to them in the future (Corcoran *et al.* 2006; Bentall *et al.* 2008, 2009; Freeman *et al.* 2013). At each entry point, participants were asked to think of what might happen in the next few hours and to rate the item ‘I think that something unpleasant will happen’ on a 7-point Likert scale (1, ‘not at all’; 7 ‘very much’). We found good concurrent validity of the ESM threat anticipation item with anxious mood (*r* = 0.49, *p* < 0.001), which has been previously reported to be closely linked to threat anticipation (Freeman *et al.* 2013).
Psychotic experiences	The ESM psychosis measure was used to assess intensity of psychotic experiences. It consists of eight items (i.e. ‘I feel paranoid’, ‘I feel unreal’, ‘I hear things that aren't really there’, ‘I see things that aren't really there’, ‘I can't get these thoughts out of my head’, ‘My thoughts are influenced by others’, ‘It's hard to express my thoughts in words’, ‘I feel like I am losing control’) rated on a 7-point Likert scale (Cronbach's *α* = 0.90) (Myin-Germeys *et al.* 2005; Palmier-Claus *et al.* 2011). We observed good concurrent validity of ESM measures of negative affect and psychotic experiences (*r* = 0.68, *p* < 0.001)

a ESM procedure: On each day over an assessment period of six consecutive days, the PsyMate^®^ emitted ten ‘beep’ signals at random moments within set blocks of time. During an initial briefing session, participants were asked to stop their activity and answer questions about thoughts, feelings, behaviours, social situations, and neighbourhood surroundings each time the device emitted the beep signal. The ESM questionnaire was available to participants for the duration of 10 min after emission of the beep signal. Participants were contacted at least once during the assessment period to assess their adherence to instructions, identify any potential distress associated with the method, and maximize the number of observations per participant. At the end of the assessment period, participants' reactivity to, and compliance with, the method was examined in a debriefing session. Participants were required to provide valid responses to at least one-third of the emitted beeps to be included in the analysis (Delespaul *et al.* 2002).

### Statistical analysis

ESM data have a multilevel structure, such that multiple observations are nested within participants. In two-level, linear mixed models, multiple observations (level 1) were treated as nested within participants (level 2) using the ‘xtmixed’ command in Stata v. 13 (StataCorp., 2013). We fitted models with each putative psychological mechanism (event-related stress, social stress, area-related stress, experiences of outsider status, threat anticipation) as the continuous independent variable and (i) negative affect and (ii) psychotic experiences as the outcome variable, while controlling for potential confounders (i.e. age, gender, ethnicity, level of education, employment status). We added two-way (mechanism × abuse, mechanism × group, abuse × group) and three-way (mechanism × abuse × group) interactions to test whether associations between psychological mechanisms and (i) negative affect and (ii) psychotic experiences were modified by prior exposure to childhood abuse (continuous CTQ sexual, physical, and emotional abuse mean scores) and group (FEP, ARMS, controls). Likelihood ratio tests were used to evaluate improvement in model fit as well as the ‘lincom’ command to compute linear combinations of coefficients for testing the hypotheses that: (1) within each group, the magnitude of associations of each mechanism with (i) negative affect and (ii) psychotic experiences was greater in individuals exposed to high *v.* low levels of childhood abuse (mean ± 1 s.d. of continuous CTQ scores) (Aiken & West, 1991; Cohen *et al.* 2003) (H1); and (2) the difference in magnitude of associations of each mechanism with (i) negative affect and (ii) psychotic experiences in those exposed to high *v.* low levels of abuse was greater in (*a*) FEP than in controls, (*b*) ARMS than in controls, and (*c*) FEP than in ARMS (H2). We standardized continuous ESM and CTQ variables (mean = 0, s.d. = 1) for interpreting significant three-way interaction terms (Dawson & Richter, 2006) and adjusted significance levels of likelihood ratio tests for three-way interactions to correct for Type-I error proliferation using family-wise error-corrected *p* values (*p*_FWE_) computed by multiplying the unadjusted *p* value by the total number of tests.

## Results

### Basic sample characteristics

During the study period, a total of 146 participants (50 FEP individuals, 44 ARMS individuals, and 52 controls) completed the CTQ and ESM assessment (with ⩾20 valid responses). ARMS and FEP individuals were younger, more often unemployed and educated to school level than controls (see Supplementary Table S1). FEP individuals reported on average higher levels of sexual [*B* = 0.32, 95% confidence interval (CI) 0.003–0.64, *p* = 0.048], physical (*B* = 0.34, 95% CI 0.05–0.63, *p* = 0.022), and emotional (*B* = 0.53, 95% CI 0.17–0.88, *p* = 0.004) abuse than controls. Levels of sexual abuse were similar in ARMS individuals and controls (*B* = 0.14, 95% CI −0.20 to 0.47, *p* = 0.422). However, physical (*B* = 0.45, 95% CI 0.15–0.75, *p* = 0.004) and emotional (*B* = 1.06, 95% CI 0.70–1.43, *p* < 0.001) abuse levels were markedly elevated in ARMS individuals. While there was no evidence of marked differences in sexual (*B* = 0.19, 95% CI −0.15 to 0.47, *p* = 0.268) and physical (*B* = −0.11, 95% CI −0.41 to 0.19, *p* = 0.476) abuse across FEP and ARMS individuals, FEP individuals reported markedly lower levels of emotional abuse (*B* = −0.54, 95% CI −0.90 to −0.17, *p* = 0.004) than ARMS individuals. In the ARMS group, 17 individuals had a SCID diagnosis of anxiety (*n* = 12), mood (*n* = 2), or mood and anxiety (*n* = 3) disorder.

### Psychological mechanisms underlying sexual abuse in FEP, ARMS, and controls

As can be seen in Table 3, we found no evidence that the association between event-related stress and (i) negative affect and (ii) psychotic experiences was modified by prior exposure to childhood sexual abuse in FEP, ARMS, and controls. However, there was strong evidence for interaction effects (all *p*_FWE_ < 0.05) of social stress × sexual abuse × group, area-related stress × sexual abuse × group, outsider status × sexual abuse × group, and threat anticipation × sexual abuse × group on (i) negative affect and (ii) psychotic experiences. These indicated that the magnitude of associations of each psychological mechanism with (i) negative affect and (ii) psychotic experiences differed between high and low levels of abuse within (H1) and across (H2) groups as detailed in the following.

**Table 3.** Psychological mechanisms underlying sexual abuse in FEP, ARMS, and controls^a^

	FEP		ARMS		Controls		LR test for interaction^b^	
	adj. *β* (95% CI)	*p*	adj. *β* (95% CI)	*p*	adj. *β* (95% CI)	*p*	χ^2^ (df)	*p*_FWE_
	Outcome: Negative affect							
Event-related stress × sexual abuse × group^c^							4.1 (2)	1.0
Social stress × sexual abuse × group^c^							13.3 (2)	0.0348
Level of sexual abuse								
High (mean + 1 s.d.)	0.29 (0.23 to 0.35)	<0.001	0.37 (0.31 to 0.43)	<0.001	0.21 (0.13 to 0.29)	<0.001		
Average (mean)	0.24 (0.19 to 0.28)	<0.001	0.36 (0.31 to 0.40)	<0.001	0.28 (0.24 to 0.33)	<0.001		
Low (mean − 1 s.d.)	0.18 (0.11 to 0.24)	<0.001	0.35 (0.28 to 0.41)	<0.001	0.35 (0.29 to 0.42)	<0.001		
High *v.* low^b^	0.11 (0.03 to 0.19)	0.005	0.02 (−0.07 to 0.12)	0.636	−0.14 (−0.25 to −0.03)	0.013		
Area-related stress × sexual abuse × group^c^							18.9 (2)	0.0021
Level of sexual abuse								
High (mean + 1 s.d.)	0.29 (0.21 to 0.36)	<0.001	0.22 (0.11 to 0.33)	<0.001	0.07 (−0.03 to 0.17)	0.153		
Average (mean)	0.19 (0.13 to 0.25)	<0.001	0.25 (0.18 to 0.31)	<0.001	0.16 (0.10 to 0.22)	<0.001		
Low (mean − 1 s.d.)	0.09 (0.01 to 0.17)	0.035	0.27 (0.17 to 0.37)	<0.001	0.24 (0.16 to 0.33)	<0.001		
High *v.* low^b^	0.20 (0.10 to 0.31)	<0.001	−0.05 (−0.21 to 0.12)	0.576	−0.17 (−0.31 to −0.03)	0.019		
Outsider status × sexual abuse × group^c^							29.5 (2)	<0.001
Level of sexual abuse								
High (mean + 1 s.d.)	0.64 (0.56 to 0.71)	<0.001	0.54 (0.47 to 0.60)	<0.001	0.19 (0.005 to 0.38)	0.045		
Average (mean)	0.50 (0.44 to 0.55)	<0.001	0.57 (0.51 to 0.62)	<0.001	0.34 (0.25 to 0.42)	<0.001		
Low (mean − 1 s.d.)	0.36 (0.28 to 0.43)	<0.001	0.60 (0.52 to 0.68)	<0.001	0.48 (0.36 to 0.60)	<0.001		
High *v.* low^b^	0.28 (0.18 to 0.38)	<0.001	−0.06 (−0.16 to 0.04)	0.238	−0.29 (−0.56 to −0.02)	0.037		
	Outcome: Psychotic experiences							
Event-related stress × sexual abuse × group^c^							6.8 (2)	0.8991
Social stress × sexual abuse × group^c^							13.0 (2)	0.0416
Level of sexual abuse								
High (mean + 1 s.d.)	0.10 (0.06 to 0.14)	<0.001	0.19 (0.15 to 0.23)	<0.001	0.07 (0.01 to 0.12)	0.020		
Average (mean)	0.04 (0.01 to 0.07)	0.018	0.16 (0.13 to 0.19)	<0.001	0.09 (0.06 to 0.12)	<0.001		
Low (mean − 1 s.d.)	−0.02 (−0.07 to 0.02)	0.360	0.13 (0.08 to 0.18)	<0.001	0.12 (0.07 to 0.16)	<0.001		
High *v.* low^b^	0.12 (0.07 to 0.18)	<0.001	0.06 (−0.01 to 0.13)	0.076	−0.05 (−0.13 to 0.03)	0.188		
Area-related stress × sexual abuse × group^c^							30.4 (2)	<0.001
Level of sexual abuse								
High (mean + 1 s.d.)	0.26 (0.21 to 0.31)	<0.001	0.23 (0.15 to 0.31)	<0.001	0.07 (0.002 to 0.14)	0.043		
Average (mean)	0.14 (0.10 to 0.18)	<0.001	0.20 (0.15 to 0.24)	<0.001	0.11 (0.07 to 0.15)	<0.001		
Low (mean − 1 s.d.)	0.01 (−0.05 to 0.06)	0.733	0.17 (0.10 to 0.23)	<0.001	0.15 (0.09 to 0.21)	<0.001		
High *v.* low^b^	0.25 (0.18 to 0.32)	<0.001	0.06 (−0.05 to 0.17)	0.282	−0.08 (−0.17 to 0.02)	0.113		
Outsider status × sexual abuse × group^c^							24.8 (2)	<0.001
Level of sexual abuse								
High (mean + 1 s.d.)	0.39 (0.34 to 0.45)	<0.001	0.39 (0.34 to 0.43)	<0.001	0.17 (0.04 to 0.30)	0.010		
Average (mean)	0.26 (0.23 to 0.30)	<0.001	0.35 (0.31 to 0.39)	<0.001	0.26 (0.20 to 0.31)	<0.001		
Low (mean − 1 s.d.)	0.14 (0.08 to 0.19)	<0.001	0.32 (0.26 to 0.37)	<0.001	0.34 (0.26 to 0.42)	<0.001		
High *v.* low^b^	0.26 (0.19 to 0.33)	<0.001	0.07 (0.003 to 0.14)	0.042	−0.17 (−0.36 to 0.02)	0.074		
Threat anticipation × sexual abuse × group^c^							12.8 (2)	0.0454
Level of sexual abuse								
High (mean + 1 s.d.)	0.29 (0.25 to 0.33)	<0.001	0.27 (0.23 to 0.32)	<0.001	0.11 (0.04 to 0.19)	0.004		
Average (mean)	0.25 (0.21 to 0.29)	<0.001	0.21 (0.18 to 0.25)	<0.001	0.17 (0.13 to 0.21)	<0.001		
Low (mean − 1 s.d.)	0.20 (0.15 to 0.26)	<0.001	0.15 (0.10 to 0.20)	<0.001	0.23 (0.16 to 0.29)	<0.001		
High *v.* low^b^	0.09 (0.03 to 0.14)	0.003	0.12 (0.05 to 0.19)	<0.001	−0.11 (−0.23 to −0.001)	0.047		

adj. *β*, Standardized regression coefficients [continuous independent variables were standardized (mean = 0, s.d. = 1) for interpreting significant three-way interaction terms and examining the difference in associations between high (mean + 1 s.d.), average (mean), and low (mean − 1 s.d.) levels of abuse within and across groups (FEP, ARMS, controls)]; ARMS, At-Risk Mental State for psychosis; CI, confidence interval; df, degrees of freedom; FEP, first-episode psychosis;  LR, likelihood ratio; *p*_FWE_, family-wise error-corrected *p* values were computed by multiplying the unadjusted *p* value by the total number of tests to adjust significance levels of likelihood ratio tests for three-way interactions; s.d., standard deviation.

a Adjusted for age, gender, ethnicity, level of education, and employment status.

b Difference in associations between those exposed to high *v.* low levels of sexual abuse across groups (Δ high *v.* low): 
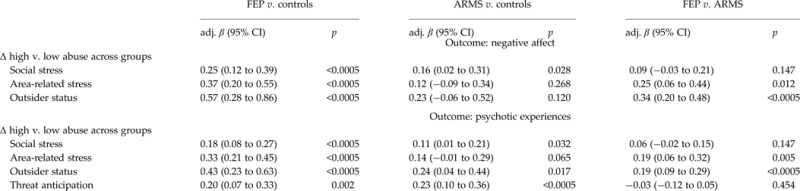

c Three-way interaction as included in the following model (with *y*_ij_ for negative affect or psychotic experiences as outcome variable): *y*_ij_ = *β*_0_ + *β*_1_(mechanism_ij_) + *β*_2_(abuse_j_) + *β*_3_(group_j_) + *β*_4_(mechanism_ij_ × abuse_j_) + *β*_5_(mechanism_ij_ × group_j_) + *β*_6_(abuse_j_ × group_j_) + *β*_7_(mechanism_ij_ × abuse_j_ × group_j_) + *ε*_ij_ (full model not shown and available upon request).

#### FEP (H1)

There was a greater association in FEP individuals exposed to high levels of sexual abuse than in FEP individuals exposed to low levels of sexual abuse between social stress (adj. *β*_high *v.* low_ = 0.11, *p* = 0.005), area-related stress (adj. *β*_high *v.* low_ = 0.20, *p* < 0.001), outsider status (adj. *β*_high *v.* low_ = 0.28, *p* < 0.001) and (i) negative affect (see Supplementary Fig. S1*a*–*c*, explanatory notes). Similarly, social stress (adj. *β*_high *v.* low_ = 0.12, *p* < 0.001), area-related stress (adj. *β*_high *v.* low_ = 0.25, *p* < 0.001), outsider status (adj. *β*_high *v.* low_ = 0.26, *p* < 0.001) and threat anticipation (adj. *β*_high *v.* low_ = 0.09, *p* = 0.003) were associated with (ii) more intense psychotic experiences in FEP individuals exposed to high *v.* low levels of sexual abuse (see Supplementary Fig. S1*d*–*g*, explanatory notes).

#### ARMS (H1)

Experiences of outsider status (adj. *β*_high *v.* low_ = 0.07, *p* = 0.042) and enhanced threat anticipation (adj. *β*_high *v.* low_ = 0.12, *p* < 0.001) were associated with more intense psychotic experiences in ARMS exposed to high levels of sexual abuse than ARMS exposed to low levels of sexual abuse. There was no evidence that the associations of other putative psychological mechanisms with (i) negative affect and (ii) psychotic experiences were greater in ARMS exposed to high *v.* low levels of sexual abuse (see Supplementary Fig. S1*a*–*g*, explanatory notes).

#### Controls (H1)

In contrast to FEP (and, in part, ARMS) individuals, in controls exposed to high levels of sexual abuse, social stress (adj. *β*_high *v.* low_ = −0.14, *p* = 0.013), area-related stress (adj. *β*_high *v.* low_ = −0.17, *p* = 0.019) and experiences of outsider status (adj. *β*_high *v.* low_ = −0.29, *p* = 0.037) were associated with less intense negative affect, as well as enhanced threat anticipation with less intense psychotic experiences (adj. *β*_high *v.* low_ = −0.11, *p* = 0.047), than in controls exposed to low levels of sexual abuse (see Supplementary Fig. S1*a*–*g*, explanatory notes).

#### Group comparison (H2)

When we examined differences in the magnitude of associations of putative psychological mechanisms with (i) negative affect and (ii) psychotic experiences between those exposed to high *v.* low levels of sexual abuse across groups, we consistently observed marked differences across FEP and controls and, less consistent and marked, ARMS and controls (see Table 3, note c; Supplementary Fig. S1, explanatory notes). So for example, there was evidence that the difference in emotional reactivity to social stress between those exposed to high *v.* low levels of sexual abuse significantly varied across FEP *v.* controls (adj. *β*_Δhigh *v.* low_ = 0.25, *p* < 0.0005) and ARMS *v.* controls (adj. *β*_Δhigh *v.* low_ = 0.16, *p* = 0.028); also, the difference in associations of social stress and psychotic experiences between those exposed to high *v.* low levels of sexual abuse across groups was greatest in FEP *v.* controls (adj. *β*_Δhigh *v.* low_ = 0.18, *p* < 0.0005), followed by ARMS *v.* controls (adj. *β*_Δhigh *v.* low_ = 0.11, *p* = 0.032). When we further compared FEP and ARMS, differences in associations of area-related stress, outsider status and (i) negative affect and (ii) psychotic experiences between those exposed to high *v.* low levels of sexual abuse were greater in FEP than in ARMS.

### Psychological mechanisms underlying physical abuse in FEP, ARMS, and controls

We found no interaction effects of event-related stress × physical abuse × group, social stress × physical abuse × group, area-related stress × physical abuse × group, and outsider status × physical abuse × group on (i) negative affect and (ii) psychotic experiences (see Table 4). However, an interaction effect of threat anticipation × physical abuse × group on psychotic experiences (see Supplementary Fig. S2) indicated that enhanced threat anticipation was associated with more intense psychotic experiences in ARMS individuals exposed to high *v.* low levels of physical abuse (adj. *β*_high *v.* low_ = 0.22, *p* < 0.001). In FEP (adj. *β*_high *v.* low_ = 0.04, *p* = 0.286) and controls (adj. *β*_high *v.* low_ = −0.03, *p* = 0.716), this association was similar in those with high *v.* low levels of physical abuse.

**Table 4.** Psychological mechanisms underlying physical abuse in FEP, ARMS, and controls^a^

	FEP		ARMS		Controls		LR test for interaction^b^	
	adj. *β* (95% CI)	*p*	adj. *β* (95% CI)	*p*	adj. *β* (95% CI)	*p*	χ^2^ (df)	*p*_FWE_
	Outcome: Negative affect							
Event-related stress × physical abuse × group^c^							7.5 (2)	0.6337
Social stress × physical abuse × group^c^							11.3 (2)	0.0926
Area-related stress × physical abuse × group^c^							5.4 (2)	1.0
Outsider status × physical abuse × group^c^							3.8 (2)	1.0
	Outcome: Psychotic experiences							
Event-related stress × physical abuse × group^c^							1.2 (2)	1.0
Social stress × physical abuse × group^c^							6.1 (2)	1.0
Area-related stress × physical abuse × group^c^							4.4 (2)	1.0
Outsider status × physical abuse × group^c^							6.8 (2)	0.9103
Threat anticipation × physical abuse × group^c^							18.0 (2)	0.0034
Level of physical abuse								
High (mean + 1 s.d.)	0.28 (0.23 to 0.33)	<0.001	0.29 (0.25 to 0.34)	<0.001	0.16 (0.07 to 0.26)	0.001		
Average (mean)	0.26 (0.22 to 0.30)	<0.001	0.19 (0.15 to 0.22)	<0.001	0.17 (0.13 to 0.22)	<0.001		
Low (mean − 1 s.d.)	0.24 (0.19 to 0.29)	<0.001	0.08 (0.03 to 0.13)	0.003	0.19 (0.12 to 0.25)	<0.001		
High *v.* low^b^	0.04 (−0.03 to 0.11)	0.286	0.22 (0.15 to 0.28)	<0.001	−0.03 (−0.16 to 0.11)	0.716		

adj. *β*, Standardized regression coefficients [continuous independent variables were standardized (mean = 0, s.d. = 1) for interpreting significant three-way interaction terms and examining the difference in associations between high (mean + 1 s.d.), average (mean), and low (mean − 1 s.d.) levels of abuse within and across groups (FEP, ARMS, controls)]; ARMS, At-Risk Mental State for psychosis; CI, confidence interval; df, degrees of freedom; FEP, first-episode psychosis;  LR, likelihood ratio; *p*_FWE_, family-wise error-corrected *p* values were computed by multiplying the unadjusted *p* value by the total number of tests to adjust significance levels of likelihood ratio tests for three-way interactions; s.d., standard deviation.

a Adjusted for age, gender, ethnicity, level of education, and employment status.

b Difference in associations between those exposed to high *v.* low levels of physical abuse across groups (Δ high *v.* low): 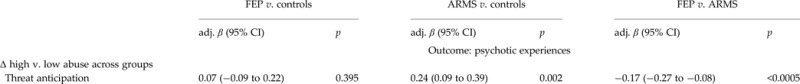

c Three-way interaction as included in the following model (with *y*_ij_ for negative affect or psychotic experiences as outcome variable): *y*_ij_ = *β*_0_ + *β*_1_(mechanism_ij_) + *β*_2_(abuse_j_) + *β*_3_(group_j_) + *β*_4_(mechanism_ij_ × abuse_j_) + *β*_5_(mechanism_ij_ × group_j_) + *β*_6_(abuse_j_ × group_j_) + *β*_7_(mechanism_ij_ × abuse_j_ × group_j_) + *ε*_ij_ (full model not shown and available upon request).

### Psychological mechanisms underlying emotional abuse in FEP, ARMS, and controls

There was no evidence that associations between event-related stress, social stress, area-related stress, outsider status and (i) negative affect and (ii) psychotic experiences were modified by childhood emotional abuse in FEP, ARMS, and controls (see Table 5). However, we found an interaction effect of threat anticipation × emotional abuse × group (see Supplementary Fig. S3). This indicated that enhanced threat anticipation was associated with more psychotic experiences in ARMS individuals exposed to high *v.* low levels of emotional abuse (adj. *β*_high *v.* low_ = 0.20, *p* < 0.001), but neither in FEP individuals (adj. *β*_high *v.* low_ = −0.03, *p* = 0.392) nor controls (adj. *β*_high *v.* low_ = 0.02, *p* = 0.709) with high and low levels of emotional abuse.

**Table 5.** Psychological mechanisms underlying emotional abuse in FEP, ARMS, and controls^a^

	FEP		ARMS		Controls		LR test for interaction^b^	
	adj. *β* (95% CI)	*p*	adj. *β* (95% CI)	*p*	adj. *β* (95% CI)	*p*	χ^2^ (df)	*p*_FWE_
	Outcome: Negative affect							
Event-related stress × emotional abuse × group^c^							9.9 (2)	0.1898
Social stress × emotional abuse × group^c^							4.7 (2)	1.0
Area-related stress × emotional abuse × group^c^							1.8 (2)	1.0
Outsider status × emotional abuse × group^c^							7.5 (2)	0.6248
	Outcome: Psychotic experiences							
Event-related stress × emotional abuse × group^c^							8.5 (2)	0.3819
Social stress × emotional abuse × group^c^							0.4 (2)	1.0
Area-related stress × emotional abuse × group^c^							7.1 (2)	0.7843
Outsider status × emotional abuse × group^c^							8.0 (2)	0.5026
Threat anticipation × emotional abuse × group^c^							22.5 (2)	0.0003
Level of emotional abuse								
High (mean + 1 s.d.)	0.24 (0.19 to 0.30)	<0.001	0.25 (0.21 to 0.28)	<0.001	0.19 (0.10 to 0.29)	<0.001		
Average (mean)	0.26 (0.22 to 0.30)	<0.001	0.15 (0.11 to 0.19)	<0.001	0.18 (0.14 to 0.23)	<0.001		
Low (mean − 1 s.d.)	0.28 (0.22 to 0.34)	<0.001	0.05 (−0.01 to 0.11)	0.106	0.17 (0.11 to 0.23)	<0.001		
High *v.* low^b^	−0.03 (−0.11 to 0.04)	0.392	0.20 (0.14 to 0.25)	<0.001	0.02 (−0.10 to 0.15)	0.709		

adj. *β*, standardized regression coefficients [continuous independent variables were standardized (mean = 0, s.d. = 1) for interpreting significant three-way interaction terms and examining the difference in associations between high (mean + 1 s.d.), average (mean), and low (mean − 1 s.d.) levels of abuse within and across groups (FEP, ARMS, controls)]; ARMS, At-Risk Mental State for psychosis; CI, confidence interval; df, degrees of freedom; FEP, first-episode psychosis; LR, likelihood ratio; *p*_FWE_, family-wise error-corrected *p* values were computed by multiplying the unadjusted *p* value by the total number of tests to adjust significance levels of likelihood ratio tests for three-way interactions; s.d., standard deviation.

a Adjusted for age, gender, ethnicity, level of education, and employment status.

b Difference in associations between those exposed to high *v.* low levels of emotional abuse across groups (Δ high *v.* low): 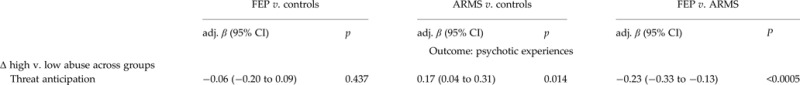

c Three-way interaction as included in the following model (with *y*_ij_ for negative affect or psychotic experiences as outcome variable): *y*_ij_ = *β*_0_ + *β*_1_(mechanism_ij_) + *β*_2_(abuse_j_) + *β*_3_(group_j_) + *β*_4_(mechanism_ij_ × abuse_j_) + *β*_5_(mechanism_ij_ × group_j_) + *β*_6_(abuse_j_ × group_j_) + *β*_7_(mechanism_ij_ × abuse_j_ × group_j_) + *ε*_ij_ (full model not shown and available upon request).

## Discussion

### Principal findings

Using an experience sampling design, this study found strong and consistent evidence that various forms of minor interpersonal and environmental stress in daily life (i.e. social stress, outsider status, area-related stress) were associated with both elevated negative affect and more intense psychotic experiences in FEP individuals exposed to high *v.* low levels of childhood sexual abuse. The association between threat anticipation and psychotic experiences was also greater in FEP individuals exposed to high *v.* low levels of sexual abuse. Our findings further suggest that, controls exposed to high levels of sexual abuse were, by contrast, more resilient, with minor socio-environmental stressors being associated with less intense negative affect and enhanced threat anticipation with less intense psychotic experiences than in controls exposed to low levels of sexual abuse. ARMS individuals formed an intermediate group, with only some evidence of more intense psychotic experiences associated with experiences of outsider status and enhanced threat anticipation in those exposed. A less clear-cut pattern emerged from findings on putative psychological mechanisms underlying physical and emotional abuse. ARMS individuals, but not FEP individuals and controls, exposed to physical and emotional abuse reported more intense psychotic experiences in relation to enhanced threat anticipation.

### Comparison with previous research

In recent years, it has been repeatedly proposed that exposure to trauma and abuse early in life may impact on the development of psychosis by increasing vulnerability to the negative effects of subsequent adversity via elevated stress sensitivity and enhanced threat anticipation as important psychological mechanisms on a socio-developmental pathway to psychosis (Morgan & Hutchinson, 2010; Morgan *et al.* 2010, 2014). However, evidence in support of this proposition remained limited. We observed a consistent pattern of findings in FEP individuals that suggests exposure to sexual abuse may sensitize individuals to the negative effects of more minor adverse social contexts and experiences later in daily life, indexed by elevated sensitivity to social stress, area-related stress, and experiences of outsider status, as a potential psychological process associated with the development of psychotic experiences. Exposure to adverse social experiences such as childhood sexual abuse, which have recently been linked to increased striatal dopamine synthesis (Egerton *et al.* 2014) and involve the quality of interpersonal violence and threat, have been posited as having a particular relevance for, and specificity to, psychotic disorders (Harris, 1987; Bebbington *et al.* 2004; Morgan & Hutchinson, 2010). Notably, exposure to childhood sexual abuse specifically increased sensitivity to interpersonal stress in daily life (i.e. unpleasant social situations, experiences of outsider status) but not event-related stress or daily hassles in our case sample. A similar pattern was evident in ARMS individuals, who reported more intense psychotic experiences in response to experiences of outsider status following exposure to high levels of sexual abuse. We may therefore speculate that one specific pathway for the impact of childhood sexual abuse on psychosis may be via heightened interpersonal sensitivity by creating an enduring sense of feeling vulnerable in the presence of others (Bell & Freeman, 2014), which has been previously reported to be a relevant psychological mechanism in individuals with a psychotic disorder and ARMS (Bell & Freeman, 2014). What is more, our findings extended beyond interpersonal sensitivity and also involved sensitivity to wider adverse socio-environmental contexts in daily life (i.e. unpleasant neighbourhoods) and, as has been previously proposed, an enduring sense of anticipating further unpleasant events and threat (Corcoran *et al.* 2006; Bentall *et al.* 2009, 2014; Morgan *et al.* 2010; Freeman *et al.* 2013).

In contrast to our first hypothesis (of greater associations in those exposed within *each* group), we observed that social stress, area-related stress and experiences of outsider status were associated with less intense negative affect, as well as enhanced threat anticipation with a lower intensity of psychotic experiences in controls exposed to high levels of sexual abuse. Also, the difference in these associations between those exposed to high and low levels of abuse were not, as predicted, greater in FEP individuals than controls, as, by contrast, associations were reversed in controls. This is a striking finding, which strongly points toward controls with prior exposure to sexual abuse being less sensitive and, in fact, more resilient to socio-environmental stress in daily life. It links in with consistent evidence that a considerable proportion of individuals exposed to sexual abuse in childhood subsequently show resilience to psychopathology and positive psychosocial functioning in adolescence and adulthood (Collishaw *et al.* 2007; Jaffee *et al.* 2007; Rutter, 2007). Good quality interpersonal relationships have previously been found to be associated with resilience to the development of adult psychopathology in individuals exposed to severe sexual or physical abuse in childhood (Collishaw *et al.* 2007). Further, a recent systematic review by Gayer-Anderson & Morgan (2013) reported increased social networks and support in controls than individuals with psychotic experiences or first-episode psychosis. Although tentative, one possibility therefore is that (better) access to, and good quality of, social networks and support may have enhanced interpersonal resilience to adverse social experiences (i.e. unpleasant social situations, experiences of outsider status) in daily life and, thereby, averted exposure to sexual abuse from exerting its detrimental effects in controls. Along similar lines, growing up in a positive home environment and low-crime, high-social cohesion neighbourhoods have been previously found to be associated with resilience in children exposed to childhood trauma (Jaffee *et al.* 2007; Rutter, 2007). There is also some evidence that resilience is associated with more rapid recovery from anticipation of threat (Tugade & Fredrickson, 2004; Waugh *et al.* 2008). Genetic moderation of resilience to the environment may potentially explain further why controls responded differently to sexual abuse than FEP individuals (Rutter, 2007). While speculative, resilience to adverse socio-environmental contexts (i.e. area-related stress) and anticipation of threat, in interaction with (lower) polygenic risk, may in part account for our findings in controls exposed to high levels of sexual abuse. Viewed this way, ARMS individuals may, then, form an intermediate group of resilient and non-resilient individuals, for whom we found only some, limited evidence of elevated sensitivity to socio-environmental stress (i.e. experiences of outsider status) in those exposed to childhood sexual abuse at a group level. Given, further, a considerable proportion of ARMS individuals experience comorbid anxiety (Fusar-Poli *et al.* 2013*b*), possibly as a result of higher levels of emotional abuse (as observed in our sample), and anxiety is commonly considered to drive increased threat anticipation (Freeman *et al.* 2013), a specific affective pathway from emotional abuse via elevated anxiety and increased threat anticipation may crystallize in this group.

### Methodological considerations

These findings should be viewed in the light of several potential methodological issues. First, we used the CTQ, a retrospective, self-report measure of childhood sexual, physical and emotional abuse. One common concern about retrospective measures of childhood trauma is that they may be susceptible to recall bias and affected by cognitive impairments or positive symptoms associated with psychotic disorder (Fisher *et al.* 2011; Susser & Widom, 2012). ESM ratings of putative psychological mechanisms and psychotic experiences were also based on subjective self-report. However, good reliability and validity has recently been reported for retrospective self-reports of early experiences obtained from individuals with a psychotic disorder (Fisher *et al.* 2011). Similarly, the ESM has been found to be a reliable and valid assessment method in individuals with ARMS and psychotic disorder in previous studies (Myin-Germeys *et al.* 2001, 2005, 2009; Palmier-Claus *et al.* 2012). This allowed us to assess psychological mechanisms and psychotic experiences in the real world and in real time. In addition, we adopted a recently suggested approach to reducing recall bias by measuring childhood trauma before the outcome of interest (i.e. psychotic disorder) in the ARMS sample (Susser & Widom, 2012) and moved beyond previous experience sampling research in restricting our sample of patients to those with a first episode of psychosis. Although not drug-naïve, this sample allowed us to minimize the impact of illness chronicity and other consequences of psychotic disorder, which may have affected findings from previous studies in patients with enduring psychosis (Lardinois *et al.* 2011). Coupled with our ARMS sample without any prior treatment with an antipsychotic for a psychotic episode, this provided evidence on childhood trauma and putative causal mechanisms prior to (i.e. during the prodromal period in (some of) the ARMS individuals) and at first onset of psychotic disorder. Second, ESM data collection is time intense and may be associated with assessment burden for participants. Therefore, we cannot rule out that selection bias may have occurred as a result of this. Third, cross-sectional modelling of experience sampling data did not allow us to systematically examine temporal priority of putative psychological mechanisms over psychotic experiences or other criteria for establishing causal relations. We therefore cannot rule out that the differences across groups may be explained by the different stages of early psychosis. Fourth, while the prevalence of sexual abuse was similar to what has been previously reported (Fisher *et al.* 2009; Pereda *et al.* 2009; Thompson *et al.* 2014), the number of participants reporting moderate or severe abuse was, in absolute terms, still relatively small (see Supplementary Table S1). This did not allow for probing findings further, for example, with regard to potential gender differences that may have operated on putative psychological mechanisms given the prevalence and impact of childhood trauma on later psychopathology has been previously found to differ between men and women (e.g. Fisher *et al.* 2009; Pereda *et al.* 2009). Last, the number of tests for assessing three-way interactions that we conducted for each type of abuse and psychological mechanism may have inflated Type I error. However, we adjusted significance levels of these tests and only considered *p*_FWE_ for assessing evidence of three-way interactions. In addition, for statistically significant three-way interactions, effect sizes for the difference in associations between individuals exposed to high and low levels of abuse within and across groups were overall of small to moderate magnitude (in particular, within FEP and when comparing FEP and controls), which reflects a substantial, cumulative impact of abuse on putative psychological mechanisms in daily life.

## Conclusions

Our findings suggest that enhanced threat anticipation and elevated sensitivity to socio-environmental stress in daily life are important psychological processes underlying the association between childhood sexual abuse and psychosis. Some initial evidence of specificity emerged for the impact of socio-environmental exposures involving the quality of interpersonal threat such as childhood sexual abuse via pathways through heightened interpersonal sensitivity in daily life. At the same time, findings in our control sample tentatively suggest interpersonal resilience and, more broadly, resilience to adverse social contexts may potentially take on the role of protective factors associated with the development of psychotic experiences. More generally, this supports the proposition that specific risk and protective factors of psychosis emerge over time, with distal factors exerting their effects by increasing vulnerability or resilience to the effects of more proximal exposures via specific psychological mechanisms. We now need to develop and evaluate ecological momentary interventions that directly target these mechanisms and reduce the intensity of psychotic experiences in daily life (Reininghaus *et al.* 2016*b*), with the goal of promoting resilience to, and preventing onset of, psychosis.

## Supplementary material

For supplementary material accompanying this paper visit http://dx.doi.org/10.1017/S003329171600146X. click here to view supplementary material
