# Supplementary material for: Psychological processes underlying the association between childhood trauma and psychosis in daily life: an experience sampling study
Source: Psychol Med. 2016 Jul 12;46(13):2799–813. doi: 10.1017/S003329171600146X (PMC5358473; doi:10.1017/S003329171600146X)
Supplement: Supplementary file 1 [file S003329171600146Xsup.zip › S003329171600146Xsup001.docx]

**SUPPLEMENTARY FIGURES**

**Supplementary Figure S1.** Psychological mechanisms underlying sexual abuse in FEP, ARMS, and controls

**Supplementary Figure S1*a*.** Association between social stress and negative affect at high (mean+1 SD), average (mean), and low (mean-1 SD) levels of sexual abuse in FEP, ARMS, and controls^a,b^

**FEP ARMS**  **Controls**

*Note:* FEP, First-Episode Psychosis; ARMS, At-Risk Mental State for psychosis; SD, standard deviation; df, degrees of freedom; p_fwe_, family-wise error-corrected p-values; vs., versus

^a^ Adjusted for age, gender, ethnicity, level of education, and employment status

^b^ Social stress × sexual abuse × group interaction, χ^2^=13.3, df=2, p_fwe_=0.0348; association between social stress and negative affect in each group by abuse:

|  | | *FEP* | |  | *ARMS* | |  | *Controls* | |
| --- | --- | --- | --- | --- | --- | --- | --- | --- | --- |
|  | | adj. β (95% CI) | P |  | adj. β (95% CI) | p |  | adj. β (95% CI) | p |
| CTQ sexual abuse | |  |  |  |  |  |  |  |  |
|  | High (mean+1 SD) | 0.29 (0.23 – 0.35) | <0.001 |  | 0.37 (0.31 – 0.43) | <0.001 |  | 0.21 (0.13 – 0.29) | <0.001 |
|  | Average (mean) | 0.24 (0.19 – 0.28) | <0.001 |  | 0.36 (0.31 – 0.40) | <0.001 |  | 0.28 (0.24 – 0.33) | <0.001 |
|  | Low (mean-1 SD) | 0.18 (0.11 – 0.24) | <0.001 |  | 0.35 (0.28 – 0.41) | <0.001 |  | 0.35 (0.29 – 0.42) | <0.001 |
|  | High vs. low | 0.11 (0.03 – 0.19) | 0.005 |  | 0.02 (-0.07 – 0.12) | 0.636 |  | -0.14 (-0.25 – -0.03) | 0.013 |

^c^ **Explanatory Note:** The association between social stress and negative affect was greater in FEP individuals exposed to high levels of sexual abuse than in FEP individuals exposed to low levels of sexual abuse (adj. β_high vs. low_=0.11, p=0.005). By contrast, compared with controls exposed to low levels of sexual abuse, those exposed to high levels of sexual abuse reported less intense negative emotional reactions to social stress (adj. β _high vs. low_=-0.14, p=0.013). The association between social stress and negative affect was similar in ARMS with high and low levels of sexual abuse (adj. β_high vs. low_=0.02, p=0.636). There was evidence that the difference in the association between social stress and negative affect in subjects exposed to high vs. low levels of sexual abuse significantly varied across (and was reversed in) FEP vs. controls (adj. β_Δ high vs. low_=0.25, 95% CI 0.12-0.39, p<0.0005). We further found that that the difference in this association between those exposed to high vs. low levels of sexual abuse significantly varied across ARMS vs. controls (adj. β_Δ high vs. low_=0.16, 95% CI 0.02-0.31, p=0.028) but not FEP vs. ARMS (adj. β_Δ high vs. low_=0.09, 95% CI -0.03-0.21, p=0.147).

**Supplementary Figure S1*b*.** Association between area-related stress and negative affect at high (mean+1 SD), average (mean), and low (mean-1 SD) levels of sexual abuse in FEP, ARMS, and controls^a,b^

**FEP ARMS Controls**

*Note:* FEP, First-Episode Psychosis; ARMS, At-Risk Mental State for psychosis; SD, standard deviation; df, degrees of freedom; p_fwe_, family-wise error-corrected p-values; vs., versus

^a^ Adjusted for age, gender, ethnicity, level of education, and employment status

^b^ Area-related stress × sexual abuse × group interaction, χ^2^=18.9, df=2, p_fwe_=0.0021; association between area-related stress and negative affect in each group by abuse:

|  | | *FEP* | |  | *ARMS* | |  | *Controls* | |
| --- | --- | --- | --- | --- | --- | --- | --- | --- | --- |
|  | | adj. β (95% CI) | p |  | adj. β (95% CI) | p |  | adj. β (95% CI) | p |
| CTQ sexual abuse | |  |  |  |  |  |  |  |  |
|  | High (mean+1 SD) | 0.29 (0.21 – 0.36) | <0.001 |  | 0.22 (0.11 – 0.33) | <0.001 |  | 0.07 (-0.03 – 0.17) | 0.153 |
|  | Average (mean) | 0.19 (0.13 – 0.25) | <0.001 |  | 0.25 (0.18 – 0.31) | <0.001 |  | 0.16 (0.10 – 0.22) | <0.001 |
|  | Low (mean-1 SD) | 0.09 (0.01 – 0.17) | 0.035 |  | 0.27 (0.17 – 0.37) | <0.001 |  | 0.24 (0.16 – 0.33) | <0.001 |
|  | High vs. low | 0.20 (0.10 – 0.31) | <0.001 |  | -0.05 (-0.21 – 0.12) | 0.576 |  | -0.17 (-0.31 – -0.03) | 0.019 |

^c^ **Explanatory Note:** The association between area-related stress and negative affect was greater in FEP individuals exposed to high levels of sexual abuse than in FEP individuals exposed to low levels of sexual abuse (adj. β_high vs. low_=0.20, p<0.001). By contrast, compared with controls exposed to low levels of sexual abuse, those exposed to high levels of sexual abuse reported less intense negative emotional reactions to area-related stress (adj. β_high vs. low_=-0.17, p=0.019). The association between area-related stress and negative affect was similar in ARMS with high and low levels of sexual abuse (adj. β_high vs. low_=-0.05, p=0.576). There was evidence that the difference in the association between area-related stress and negative affect in subjects exposed to high vs. low levels of sexual abuse significantly varied across (and was reversed in) FEP vs. controls (adj. β_Δ high vs. low_=0.37, 95% CI 0.20 – 0.55, p<0.0005). We further found that that the difference in this association between those exposed to high vs. low levels of sexual abuse significantly varied across FEP vs. ARMS (adj. β_Δ high vs. low_=0.25, 95% CI 0.06 – 0.44, p=0.012) but not ARMS vs. controls (adj. β_Δ high vs. low_=0.12, 95% CI -0.09 – 0.34, p=0.268).

**Supplementary Figure S1*c*.** Association between outsider status and negative affect at high (mean+1 SD), average (mean), and low (mean-1 SD) levels of sexual abuse in FEP, ARMS, and controls^a,b^

**FEP ARMS Controls**

*Note:* FEP, First-Episode Psychosis; ARMS, At-Risk Mental State for psychosis; SD, standard deviation; df, degrees of freedom; p_fwe_, family-wise error-corrected p-values; vs., versus

^a^ Adjusted for age, gender, ethnicity, level of education, and employment status

^b^ Outsider status × sexual abuse × group interaction, χ^2^=29.5, df=2, p_fwe_<0.001; association between outsider status and negative affect in each group by abuse:

|  | | *FEP* | |  | *ARMS* | |  | *Controls* | |
| --- | --- | --- | --- | --- | --- | --- | --- | --- | --- |
|  | | adj. β (95% CI) | P |  | adj. β (95% CI) | p |  | adj. β (95% CI) | p |
| CTQ sexual abuse | |  |  |  |  |  |  |  |  |
|  | High (mean+1 SD) | 0.64 (0.56 – 0.71) | <0.001 |  | 0.54 (0.47 – 0.60) | <0.001 |  | 0.19 (0.005 – 0.38) | 0.045 |
|  | Average (mean) | 0.50 (0.44 – 0.55) | <0.001 |  | 0.57 (0.51 – 0.62) | <0.001 |  | 0.34 (0.25 – 0.42) | <0.001 |
|  | Low (mean-1 SD) | 0.36 (0.28 – 0.43) | <0.001 |  | 0.60 (0.52 – 0.68) | <0.001 |  | 0.48 (0.36 – 0.60) | <0.001 |
|  | High vs. low | 0.28 (0.18 – 0.38) | <0.001 |  | -0.06 (-0.16 – 0.04) | 0.238 |  | -0.29 (-0.56 – -0.02) | 0.037 |

^c^ **Explanatory Note:** The association between outsider status and negative affect was greater in FEP individuals exposed to high levels of sexual abuse than in FEP individuals exposed to low levels of sexual abuse (adj. β_high vs. low_=0.28, p<0.001). By contrast, compared with controls exposed to low levels of sexual abuse, those exposed to high levels of sexual abuse reported less intense negative emotional reactions to experiences of outsider status (adj. β_high vs. low_=-0.29, p=0.037). This association was similar in ARMS (adj. β_high vs. low_=-0.06, p=0.238) with high and low levels of sexual abuse. There was evidence that the difference in the association between outsider status and negative affect in subjects exposed to high vs. low levels of sexual abuse was significantly greater in FEP vs. controls (adj. β_Δ high vs. low_=0.57, 95% CI 0.28 – 0.86, p<0.0005) and FEP vs. ARMS (adj. β_Δ high vs. low_=0.34, 95% CI 0.20 – 0.48, p<0.0005), but not ARMS vs. controls (adj. β_Δ high vs. low_=0.23, 95% CI -0.06 – 0.52, p=0.120).

**Supplementary Figure S1*d*.** Association between social stress and psychotic experiences at high (mean + 1 SD), average (mean), and low (mean - 1 SD) levels of sexual abuse in FEP, ARMS, and controls^a,b^

**FEP ARMS Controls**

*Note:* FEP, First-Episode Psychosis; ARMS, At-Risk Mental State for psychosis; SD, standard deviation; df, degrees of freedom; p_fwe_, family-wise error-corrected p-values; vs., versus

^a^ Adjusted for age, gender, ethnicity, level of education, and employment status

^b^ Social stress × sexual abuse × group interaction, χ^2^=13.0, df=2, p_fwe_=0.0416; association between social stress and psychotic experiences in each group by abuse:

|  | | *FEP* | |  | *ARMS* | |  | *Controls* | |
| --- | --- | --- | --- | --- | --- | --- | --- | --- | --- |
|  | | adj. β (95% CI) | p |  | adj. β (95% CI) | p |  | adj. β (95% CI) | p |
| CTQ sexual abuse | |  |  |  |  |  |  |  |  |
|  | High (mean+1 SD) | 0.10 (0.06 – 0.14) | <0.001 |  | 0.19 (0.15 – 0.23) | <0.001 |  | 0.07 (0.01 – 0.12) | 0.020 |
|  | Average (mean) | 0.04 (0.01 – 0.07) | 0.018 |  | 0.16 (0.13 – 0.19) | <0.001 |  | 0.09 (0.06 – 0.12) | <0.001 |
|  | Low (mean-1 SD) | -0.02 (-0.07 – 0.02) | 0.360 |  | 0.13 (0.08 – 0.18) | <0.001 |  | 0.12 (0.07 – 0.16) | <0.001 |
|  | High vs. low | 0.12 (0.07 – 0.18) | <0.001 |  | 0.06 (-0.01 – 0.13) | 0.076 |  | -0.05 (-0.13 – 0.03) | 0.188 |

^c^ **Explanatory Note:** The association between social stress and psychotic experiences was greater in FEP individuals exposed to high levels of sexual abuse than in FEP individuals exposed to low levels of sexual abuse (adj. β _high vs. low_=0.12, p<0.001). While there was some evidence that the association between social stress and psychotic experiences was also greater in ARMS exposed to high vs. low levels of sexual abuse (adj. β _high vs. low_=0.06, p=0.076), this association was similar in controls with high and low levels of sexual abuse (adj. β _high vs. low_=-0.05, p=0.188). Further, we found that the difference in the association between social stress and psychotic experiences in subjects exposed to high vs. low levels of sexual abuse was significantly greater in FEP than controls (adj. β_Δ high vs. low_=0.18, 95% CI 0.08 – 0.27, p<0.0005) as well as ARMS than controls (adj. β_Δ high vs. low_=0.11, 95% CI 0.01 – 0.21, p=0.032), but not FEP vs. ARMS (adj. β_Δ high vs. low_=0.06, 95% CI -0.02 – 0.15, p=0.147).

**Supplementary Figure S1*e*.** Association between area-related stress and psychotic experiences at high (mean+1 SD), average (mean), and low (mean-1 SD) levels of sexual abuse in FEP, ARMS, and controls^a,b^

**FEP ARMS Controls**

*Note:* FEP, First-Episode Psychosis; ARMS, At-Risk Mental State for psychosis; SD, standard deviation; df, degrees of freedom; p_fwe_, family-wise error-corrected p-values; vs., versus

^a^ Adjusted for age, gender, ethnicity, level of education, and employment status

^b^ Area-related stress × sexual abuse × group interaction, χ^2^=30.4, df=2, p_fwe_<0.001; association between area-related stress and psychotic experiences in each group by abuse:

|  | | *FEP* | |  | *ARMS* | |  | *Controls* | |
| --- | --- | --- | --- | --- | --- | --- | --- | --- | --- |
|  | | adj. β (95% CI) | p |  | adj. β (95% CI) | p |  | adj. β (95% CI) | p |
| CTQ sexual abuse | |  |  |  |  |  |  |  |  |
|  | High (mean+1 SD) | 0.26 (0.21 – 0.31) | <0.001 |  | 0.23 (0.15 – 0.31) | <0.001 |  | 0.07 (0.002 – 0.14) | 0.043 |
|  | Average (mean) | 0.14 (0.10 – 0.18) | <0.001 |  | 0.20 (0.15 – 0.24) | <0.001 |  | 0.11 (0.07 – 0.15) | <0.001 |
|  | Low (mean-1 SD) | 0.01 (-0.05 – 0.06) | 0.733 |  | 0.17 (0.10 – 0.23) | <0.001 |  | 0.15 (0.09 – 0.21) | <0.001 |
|  | High vs. low | 0.25 (0.18 – 0.32) | <0.001 |  | 0.06 (-0.05 – 0.17) | 0.282 |  | -0.08 (-0.17 – 0.02) | 0.113 |

^c^ **Explanatory Note:** The association between area-related stress and psychotic experiences was greater in FEP individuals exposed to high levels of sexual abuse than in FEP individuals exposed to low levels of sexual abuse (adj. β_high vs. low_=0.25, p<0.001). However, this association was similar in ARMS (adj. β_high vs. low_=0.06, p=0.282) and controls (adj. β_high vs. low_=-0.08, p=0.113) with high and low levels of sexual abuse. There was evidence that the difference in the association between area-related stress and psychotic experiences in subjects exposed to high vs. low levels of sexual abuse was significantly greater in FEP vs. controls (adj. β_Δ high vs. low_=0.33, 95% CI 0.21 – 0.45, <0.0005) and FEP vs. ARMS (adj. β_Δ high vs. low_=0.19, 95% CI 0.06 – 0.32, p=0.005), but not ARMS vs. controls (adj. β_Δ high vs. low_=0.14, 95% CI -0.01 – 0.29, p=0.065).

**Supplementary Figure S1*f*.** Association between outsider status and psychotic experiences at high (mean+1 SD), average (mean), and low (mean-1 SD) levels of sexual abuse in FEP, ARMS, and controls^a,b^

**FEP ARMS Controls**

*Note:* FEP, First-Episode Psychosis; ARMS, At-Risk Mental State for psychosis; SD, standard deviation; df, degrees of freedom; p_fwe_, family-wise error-corrected p-values; vs., versus

^a^ Adjusted for age, gender, ethnicity, level of education, and employment status

^b^ Outsider status × sexual abuse × group interaction, χ^2^=24.8, df=2, p_fwe_<0.001; association between outsider status and psychotic experiences in each group by abuse:

|  | | *FEP* | |  | *ARMS* | |  | *Controls* | |
| --- | --- | --- | --- | --- | --- | --- | --- | --- | --- |
|  | | adj. β (95% CI) | p |  | adj. β (95% CI) | p |  | adj. β (95% CI) | p |
| CTQ sexual abuse | |  |  |  |  |  |  |  |  |
|  | High (mean+1 SD) | 0.39 (0.34 – 0.45) | <0.001 |  | 0.39 (0.34 – 0.43) | <0.001 |  | 0.17 (0.04 – 0.30) | 0.010 |
|  | Average (mean) | 0.26 (0.23 – 0.30) | <0.001 |  | 0.35 (0.31 – 0.39) | <0.001 |  | 0.26 (0.20 – 0.31) | <0.001 |
|  | Low (mean-1 SD) | 0.14 (0.08 – 0.19) | <0.001 |  | 0.32 (0.26 – 0.37) | <0.001 |  | 0.34 (0.26 – 0.42) | <0.001 |
|  | High vs. low | 0.26 (0.19 – 0.33) | <0.001 |  | 0.07 (0.003 – 0.14) | 0.042 |  | -0.17 (-0.36 – 0.02) | 0.074 |

^c^ **Explanatory Note:** The association between outsider status and psychotic experiences was greater in FEP individuals exposed to high vs. low levels of sexual abuse (adj. β_high vs. low_=0.26, p<0.001) as well as ARMS exposed to high vs. low levels of sexual abuse (adj. β_high vs. low_=0.07, p=0.042). By contrast, there was some evidence that, compared with controls exposed to low levels of sexual abuse, those exposed to high levels of sexual abuse reported less intense psychotic experiences in response to experiences of outsider status (adj. β_high vs. low_=-0.17, p=0.074). There was evidence that the difference in the association between outsider status and psychotic experiences in subjects exposed to high vs. low levels of sexual abuse was significantly greater in FEP vs. controls (adj. β_Δ high vs. low_=0.43, 95% CI 0.23 – 0.63, p<0.0005), FEP vs. ARMS (adj. β_Δ high vs. low_=0.19, 95% CI 0.09 – 0.29, p<0.0005), and ARMS vs. controls (adj. β_Δ high vs. low_=0.24, 95% CI 0.04 – 0.44, p=0.017).

**Supplementary Figure S1*g*.** Association between threat anticipation and psychotic experiences at high (mean+1 SD), average (mean), and low (mean-1 SD) levels of sexual abuse in FEP, ARMS, and controls^a,b^

**FEP ARMS Controls**

*Note:* FEP, First-Episode Psychosis; ARMS, At-Risk Mental State for psychosis; SD, standard deviation; df, degrees of freedom; p_fwe_, family-wise error-corrected p-values; vs., versus

^a^ Adjusted for age, gender, ethnicity, level of education, and employment status

^b^ Threat anticipation × sexual abuse × group interaction, χ^2^=12.8, df=2, p_fwe_=0.0454; association between threat anticipation and psychotic experiences in each group by abuse:

|  | | *FEP* | |  | *ARMS* | |  | *Controls* | |
| --- | --- | --- | --- | --- | --- | --- | --- | --- | --- |
|  | | adj. β (95% CI) | p |  | adj. β (95% CI) | p |  | adj. β (95% CI) | p |
| CTQ sexual abuse | |  |  |  |  |  |  |  |  |
|  | High (mean+1 SD) | 0.29 (0.25 – 0.33) | <0.001 |  | 0.27 (0.23 – 0.32) | <0.001 |  | 0.11 (0.04 – 0.19) | 0.004 |
|  | Average (mean) | 0.25 (0.21 – 0.29) | <0.001 |  | 0.21 (0.18 – 0.25) | <0.001 |  | 0.17 (0.13 – 0.21) | <0.001 |
|  | Low (mean-1 SD) | 0.20 (0.15 – 0.26) | <0.001 |  | 0.15 (0.10 – 0.20) | <0.001 |  | 0.23 (0.16 – 0.29) | <0.001 |
|  | High vs. low | 0.09 (0.03 – 0.14) | 0.003 |  | 0.12 (0.05 – 0.19) | <0.001 |  | -0.11 (-0.23 – -0.001) | 0.047 |

^c^ **Explanatory Note:** The association between threat anticipation and psychotic experiences was greater in FEP individuals exposed to high vs. low levels of sexual abuse (adj. β_high vs. low_=0.09, p=0.003) as well as ARMS exposed to high vs. low levels of sexual abuse (adj. β_high vs. low_=0.12, p<0.001). By contrast, compared with controls exposed to low levels of sexual abuse, those exposed to high levels of sexual abuse reported less intense psychotic experiences in response to threat anticipation (adj. β_high vs. low_=-0.11, p=0.047). There was evidence that the difference in the association between threat anticipation and psychotic experiences in subjects exposed to high vs. low levels of sexual abuse significantly varied across (and was reversed in) FEP vs. controls (adj. β_Δ high vs. low_=0.20, 95% CI 0.07 – 0.33, p=0.002) and ARMS vs. controls (adj. β_Δ high vs. low_=0.23, 95% CI 0.10 – 0.36, p<0.0005), but not FEP vs. ARMS (adj. β_Δ high vs. low_=-0.03, 95% CI -0.12 – 0.05, p=0.456).

**Supplementary Figure S2.** Psychological mechanisms underlying physical abuse in FEP, ARMS, and controls. Association between threat anticipation and psychotic experiences at high (mean+1 SD), average (mean), and low (mean-1 SD) levels of physical abuse in FEP, ARMS, and controls^a,b^

**FEP ARMS Controls**

*Note:* FEP, First-Episode Psychosis; ARMS, At-Risk Mental State for psychosis; SD, standard deviation; df, degrees of freedom; p_fwe_, family-wise error-corrected p-values; vs., versus

^a^ Adjusted for age, gender, ethnicity, level of education, and employment status

^b^ Threat anticipation × physical abuse × group interaction, χ^2^=18.0, df=2, p_fwe_=0.0034; association between threat anticipation and psychotic experiences in each group by abuse:

|  | | *FEP* | |  | *ARMS* | |  | *Controls* | |
| --- | --- | --- | --- | --- | --- | --- | --- | --- | --- |
|  | | adj. β (95% CI) | p |  | adj. β (95% CI) | p |  | adj. β (95% CI) | p |
| CTQ physical abuse | |  |  |  |  |  |  |  |  |
|  | High (mean+1 SD) | 0.28 (0.23 – 0.33) | <0.001 |  | 0.30 (0.25 – 0.34) | <0.001 |  | 0.16 (0.07 – 0.26) | 0.001 |
|  | Average (mean) | 0.26 (0.22 – 0.30) | <0.001 |  | 0.19 (0.15 – 0.22) | <0.001 |  | 0.17 (0.13 – 0.22) | <0.001 |
|  | Low (mean-1 SD) | 0.24 (0.19 – 0.29) | <0.001 |  | 0.08 (0.03 – 0.13) | 0.003 |  | 0.19 (0.12 – 0.25) | <0.001 |
|  | High vs. low | 0.04 (-0.03 – 0.11) | 0.286 |  | 0.22 (0.15 – 0.28) | <0.001 |  | -0.03 (-0.16 – 0.11) | 0.716 |

^c^ **Explanatory Note:** The association between threat anticipation and psychotic experiences was greater in ARMS exposed to high vs. low levels of physical abuse (adj. β_high vs. low_=0.22, p<0.001). However, this association was similar in FEP individuals (adj. β_high vs. low_=0.04, p=0.286) and controls (adj. β_high vs. low_=-0.03, p=0.716) with high and low levels of sexual abuse. There was evidence that the difference in the association between threat anticipation and psychotic experiences in subjects exposed to high vs. low levels of sexual abuse significantly varied across ARMS vs. controls (adj. β_Δ high vs. low_=0.24, 95% CI 0.09 – 0.39, p=0.002) and FEP vs. ARMS (adj. β_Δ high vs. low_=-0.17, 95% CI -0.27 – -0.08, p<0.0005), but not FEP vs. controls (adj. β_Δ high vs. low_=0.07, 95% CI -0.09 – 0.22, p=0.395).

**Supplementary Figure S3.** Psychological mechanisms underlying emotional abuse in FEP, ARMS, and controls. Association between threat anticipation and psychotic experiences at high (mean+1 SD), average (mean), and low (mean-1 SD) levels of emotional abuse in FEP, ARMS; and controls^a,b^

**FEP ARMS Controls**

*Note:* FEP, First-Episode Psychosis; ARMS, At-Risk Mental State for psychosis; SD, standard deviation; df, degrees of freedom; p_fwe_, family-wise error-corrected p-values; vs., versus

^a^ Adjusted for age, gender, ethnicity, level of education, and employment status

^b^ Threat anticipation × emotional abuse × group interaction, χ^2^=22.5, df=2, p_fwe_=0.0003; association between threat anticipation and psychotic experiences in each group by abuse:

|  | | *FEP* | |  | *ARMS* | |  | *Controls* | |
| --- | --- | --- | --- | --- | --- | --- | --- | --- | --- |
|  | | adj. β (95% CI) | p |  | adj. β (95% CI) | p |  | adj. β (95% CI) | p |
| CTQ emotional abuse | |  |  |  |  |  |  |  |  |
|  | High (mean+1 SD) | 0.24 (0.19 – 0.30) | <0.001 |  | 0.25 (0.21 – 0.28) | <0.001 |  | 0.19 (0.10 – 0.29) | <0.001 |
|  | Average (mean) | 0.26 (0.22 – 0.30) | <0.001 |  | 0.15 (0.11 – 0.19) | <0.001 |  | 0.18 (0.14 – 0.23) | <0.001 |
|  | Low (mean-1 SD) | 0.28 (0.22 – 0.34) | <0.001 |  | 0.05 (-0.01 – 0.11) | 0.106 |  | 0.17 (0.11 – 0.23) | <0.001 |
|  | High vs. low | -0.03 (-0.11 – 0.04) | 0.392 |  | 0.20 (0.14 – 0.25) | <0.001 |  | 0.02 (-0.10 – 0.15) | 0.709 |

^c^ **Explanatory Note:** The association between threat anticipation and psychotic experiences was greater in ARMS exposed to high vs. low levels of emotional abuse (adj. β_high vs. low_=0.20, p<0.001). However, this association was similar in FEP individuals (adj. β_high vs. low_=-0.03, p=0.392) and controls (adj. β_high vs. low_=0.02, p=0.709) with high and low levels of sexual abuse. There was evidence that the difference in the association between threat anticipation and psychotic experiences in subjects exposed to high vs. low levels of sexual abuse significantly varied across ARMS vs. controls (adj. β_Δ high vs. low_=0.17, 95% CI 0.04 – 0.31, p=0.014) and FEP vs. ARMS (adj. β_Δ high vs. low_=-0.23, 95% CI -0.33 – -0.13, p<0.0005), but not FEP vs. controls (adj. β_Δ high vs. low_=-0.06, 95% CI -0.20 – 0.09, p=0.437).
