# Supplementary material for: Psychological processes underlying the association between childhood trauma and psychosis in daily life: an experience sampling study
Source: Psychol Med. 2016 Jul 12;46(13):2799–813. doi: 10.1017/S003329171600146X (PMC5358473; doi:10.1017/S003329171600146X)
Supplement: Supplementary file 1 [file S003329171600146Xsup.zip › S003329171600146Xsup002.docx]

**Supplementary Table S1.** *Basic sample characteristics*

|  | | FEP  (n=50) | ARMS  (n=44) | Controls  (n=52) | Test statistic | p |
| --- | --- | --- | --- | --- | --- | --- |
| Age (years), mean (SD) | | 28.4 (8.6) | 23.8 (4.7) | 34.4 (12.0) | F=16.4, df=2 | <0.001 |
| Gender, n (%) | |  |  |  |  |  |
|  | Men | 28 (56.0) | 20 (45.5) | 24 (46.2) | χ^2^=1.4, df=2 | 0.506 |
|  | Women | 22 (44.0) | 24 (54.6) | 28 (53.9) |  |  |
| Ethnicity, n (%) | |  |  |  |  |  |
|  | White British | 14 (28.0) | 15 (34.1) | 24 (46.2) | χ^2^=12.8, df=10 | 0.237 |
|  | Black African | 16 (32.0) | 7 (15.9) | 8 (15.4) |  |  |
|  | Black Caribbean | 11 (22.0) | 7 (15.9) | 6 (11.5) |  |  |
|  | Asian | 1 (2.0) | 1 (2.3) | 3 (5.8) |  |  |
|  | White Other | 4 (8.0) | 5 (11.4) | 5 (9.6) |  |  |
|  | Other | 4 (8.0) | 9 (20.5) | 6 (11.5) |  |  |
| Place of birth, n (%) | |  |  |  |  |  |
|  | UK-born | 31 (62.0) | 32 (72.7) | 32 (61.5) | χ^2^=1.6, df=2 | 0.443 |
|  | Non-UK-born | 19 (38.0) | 12 (27.3) | 20 (38.5) |  |  |
| Level of education, n (%) | |  |  |  |  |  |
|  | School | 17 (34.0) | 12 (27.9) | 8 (15.4) | χ^2^=24.2, df=4 | <0.001 |
|  | Further | 24 (48.0) | 23 (53.5) | 14 (26.9) |  |  |
|  | Higher | 9 (18.0) | 8 (18.6) | 30 (57.7) |  |  |
| Employment status, n (%) | |  |  |  |  |  |
|  | Unemployed | 29 (58.0) | 14 (31.8) | 5 (9.6) | χ^2^=27.1, df=2 | <0.001 |
|  | Other | 21 (42.0) | 30 (68.2) | 47 (90.4) |  |  |
| CTQ mean scores^a^, mean (SD) | |  |  |  |  |  |
|  | Sexual abuse | 1.59 (1.04) | 1.40 (0.69) | 1.27 (0.66) | F=2.0, df=2 | 0.139 |
|  | Physical abuse | 1.60 (0.84) | 1.70 (0.85) | 1.25 (0.49) | F=4.9, df=2 | 0.009 |
|  | Emotional abuse | 2.07 (0.92) | 2.60 (1.10) | 1.54 (0.66) | F=16.7, df=2 | <0.001 |
| OPCRIT Psychotic disorder diagnosis^b,c^, n (%) | |  |  |  |  |  |
|  | Schizophrenia | 14 (29.8) | – | – | – | – |
|  | Delusional disorder | 3 (6.4) | – | – |  |  |
|  | Schizoaffective disorder | 3 (6.4) | – | – |  |  |
|  | Manic psychosis | 7 (14.9) | – | – |  |  |
|  | Depressive psychosis | 7 (14.9) | – | – |  |  |
|  | Psychotic disorder NOS | 13 (27.7) | – | – |  |  |
| Current SCID comorbid affective disorder diagnosis^d^, n (%) | |  |  |  |  |  |
|  | Mood disorder | – | 5 (11.4) | – | – | – |
|  | Anxiety disorder^e^ | – | 13 (29.5) | – |  |  |
|  | Mood and anxiety disorder | – | 3 (6.8) | – |  |  |

*Note:* FEP, First-Episode Psychosis; ARMS, At-Risk Mental State for psychosis; SD, standard deviation; df, degrees of freedom;

^a^ Categorical CTQ severity scores by group:

|  | | FEP | ARMS | Controls | Test statistic | p |
| --- | --- | --- | --- | --- | --- | --- |
|  | | n (%) | n (%) | n (%) |  |  |
| Sexual abuse | |  |  |  |  |  |
|  | Severe to extreme | 8 (16.0) | 4 (9.1) | 3 (5.8) | χ^2^=8.9, df=6 | 0.179 |
|  | Moderate to severe | 7 (14.0) | 10 (22.7) | 5 (9.6) |  |  |
|  | Mild to moderate | 10 (20.0) | 4 (9.1) | 8 (15.4) |  |  |
|  | None | 25 (50.0) | 26 (59.1) | 36 (69.2) |  |  |
| Physical abuse | |  |  |  |  |  |
|  | Severe to extreme | 8 (16.0) | 9 (20.5) | 2 (3.9) | χ^2^=16.1, df=6 | 0.013 |
|  | Moderate to severe | 2 (4.0) | 6 (13.6) | 3 (5.8) |  |  |
|  | Mild to moderate | 8 (16.0) | 4 (9.1) | 2 (3.9) |  |  |
|  | None | 32 (64.0) | 25 (56.8) | 45 (86.5) |  |  |
| Emotional abuse | |  |  |  |  |  |
|  | Severe to extreme | 8 (16.0) | 17 (38.6) | 2 (3.85) | χ^2^=32.9, df=6 | <0.001 |
|  | Moderate to severe | 7 (14.0) | 6 (13.6) | 0 (0.0) |  |  |
|  | Mild to moderate | 11 (22.0) | 10 (22.7) | 14 (26.9) |  |  |
|  | None | 24 (48.0) | 11 (25.0) | 36 (69.2) |  |  |

^b^ Missing values: 3

^c^ DSM-IV diagnoses of psychotic disorder were determined based on structured examination of case records using the Operational CRITeria (OPCRIT) system (McGuffin *et al.*, 1991; Reininghaus *et al.*, 2016-c) as part of the “Functional Enviromics” work package of EU-GEI (EU-GEI, 2014);

^c^ Current comorbid affective disorders were assessed with the SCID (First *et al.*, 2002) as part of the “G × E Prodrome” work package of EU-GEI (EU-GEI, 2014);

^e^ PTSD, n=1
